# Retraction Note: Childhood obesity risk increases with increased screen time: a systematic review and dose–response meta-analysis

**DOI:** 10.1186/s41043-023-00432-z

**Published:** 2023-08-22

**Authors:** Andrés Alexis Ramírez-Coronel, Wamaungo Juma Abdu, Shadia Hamoud Alshahrani, Mark Treve, Abduladheem Turki Jalil, Ameer S. Alkhayyat, Nermeen Singer

**Affiliations:** 1 Catholic University of Cuenca, Azogues Campus, Azogues, Ecuador; 2 University of Palermo, Buenos Aires, Argentina; 3 National University of Education, Azogues, Ecuador; 4 CES University, Medellín, Colombia; 5 EDURES Global Link, Majalengka, West Java Indonesia; 6 Medical Surgical Nursing Department, King Khalid University, Khamis Mushate, Almahala, Saudi Arabia; 7 School of Languages and General Education, Walailak University, Nakhon Si Thammarat, Thailand; 8 Medical Laboratories Techniques Department, Al-Mustaqbal University College, Babylon, Hilla, 51001 Iraq; 9 Medical Laboratory Technology Department, College of Medical Technology, The Islamic University, Najaf, Iraq; 10 Department of Media and Children’s Culture, Ain Shams University, Cairo, Egypt

## Retraction Note: Journal of Health, Population and Nutrition (2023) 42:5 https://doi.org/10.1186/s41043-022-00344-4

The Editor-in-Chief has retracted this article because it contains material that substantially overlaps with the following article [1].

Wamaungo Juma Abdu, Shadia Hamoud Alshahrani, Andrés Alexis Ramírez-Coronel and Mark Treve do not agree to this retraction. Nermeen Singer and Abduladheem Turki Jalil have not explicitly stated whether they agree to this retraction notice. Ameer S. Alkhayyat has not responded to any correspondence from the editor about this retraction.

## References

[1] Haghjoo P, Siri G, Soleimani E (2022). Screen time increases overweight and obesity risk among adolescents: a systematic review and dose-response meta-analysis. BMC Prim Care.

